# Hyaluronidase injection for the treatment of eyelid edema: a retrospective analysis of 20 patients

**DOI:** 10.1186/2047-783X-19-30

**Published:** 2014-05-28

**Authors:** Said Hilton, Holger Schrumpf, Bettina Alexandra Buhren, Edwin Bölke, Peter Arne Gerber

**Affiliations:** 1 Medical Skin Center, Dusseldorf, Germany; 2 Department of Dermatology, Heinrich-Heine University Dusseldorf, Medical Faculty, Moorenstr 5, D-40225 Dusseldorf, Germany; 3 Department of Radiation Oncology, Heinrich-Heine University Dusseldorf, Medical Faculty, Dusseldorf, Germany

## Abstract

**Background:**

Hyaluronidase (Hylase Dessau®) is a hyaluronic acid-metabolizing enzyme, which has been shown to loosen the extracellular matrix, thereby improving the diffusion of local anesthetics. Lower eyelid edema is a common post-interventional complication of cosmetic procedures performed in the lid region, such as the injection of hyaluronic acid fillers for tear-trough augmentation. The purpose of this study was to validate the efficacy of hyaluronidase in the management of lower eyelid edema.

**Methods:**

We performed a retrospective analysis with 20 patients with lower eyelid edema. Most patients (*n* = 14) presented with edema following hyaluronic acid injection (tear-trough augmentation), whereas the minority (*n* = 6) were treated due to idiopathic edema (malar edema or malar mounds). Patients were treated by local infiltration of approximately 0.2 ml to 0.5 ml of hyaluronidase (Hylase Dessau® 20 IU to 75 IU) per eyelid. Photographs were taken prior to and seven days after infiltration.

**Results:**

Hyaluronidase was found to reduce effectively and rapidly or resolve eyelid edema after a single injection. No relevant adverse effects were observed. However, it must be noted that a hyaluronidase injection may also dissolve injected hyaluronic acid fillers and may therefore negatively affect tear-trough augmentations. While the effects of a treatment for edema due to tear-trough augmentation were permanent, malar edema and malar mounds reoccurred within two to three weeks.

**Conclusion:**

The infiltration of hyaluronidase is rapid, safe and currently the only effective option for the management of eyelid edema. No relevant adverse effects were observed.

## Background

Hyaluronic acid or hyaluronan (HA) is a non-sulfated glycosaminoglycan and is the predominant part of the skin’s extracellular matrix [1]. In humans approximately 50% of the total body HA is found in the skin [2,3]. HA has been shown to be involved in various physiological and pathological processes including angiogenesis [4], cancer progression [5], immune regulation [6] and skin aging [7,8]. The HA metabolism is controlled by specific enzymes.

Hyaluronidase is a soluble enzyme that degrades HA by hydrolyzing β 1,4-N-acetylglucosaminidic bonds [9]. In humans, six hyaluronidases have been identified (HYAL-1, -2, -3, -4, HYALP1 and PH-20) [1,10,11]. Commercial formulations of hyaluronidase are of bovine (bovine testicular hyaluronidase; e.g. Hylase Dessau®, Riemser Pharma GmbH, Greifswald, Germany) or ovine origin (ovine testicular hyaluronidase; e.g. Vitrase®, Bausch&Lomb, Rochester, NY, USA). The addition of hyaluronidase to local anesthetics has been shown to enhance safely and effectively the diffusion of the drug, thereby increasing the analgesic efficacy especially in the first minutes after injection [12,13].

Besides its use in local analgesia, the use of hyaluronidase in the office has evolved as a necessity for physicians performing soft tissue augmentations with HA-based dermal fillers [14]. Whilst volume fillers are generally safe products, rare but severe treatment-associated complications may occur. These range from the Tyndall effect, over-corrections, misplaced product or granulomas, to necrosis or even blindness due to an accidental intra-arterial injection [15,16]. Notably, these complications may occur with the application of any kind of volume filler, including HA, calcium hydroxylapatite or poly-L-lactic acid. However, to the best of our knowledge, the availability of a specific antidote (hyaluronidase) for the management of adverse effects is limited to HA-based fillers and may be one reason for the preferred use of these products [17]. Several case series and review articles report on the management of HA-filler complications using hyaluronidase [15,18-21].

The injection of HA-based fillers is an effective approach for the management of periorbital hollows (tear-trough augmentation) [22-24]. Notably, the tear-trough area is regarded as a high-risk area for the injection of volume fillers and the procedure is listed as the most challenging technique for the injection of HA-based fillers [25]. Hence, it should be performed only by experienced injectors. Retrospective analyses of the adverse effects of tear-trough augmentations with HA fillers report bruising (10 to 75%), color changes (4 to 7%), contour irregularities (11%) and swelling, fluid or edema (15 to 26%) [22-24]. Nevertheless, the development of eyelid edema, often also referred to as malar bags, malar edema or malar mounds, is not a sole consequence of esthetic procedures but is an unspecific sign, and may herald a variety of systemic or periorbital diseases. Etiologies span the fields of dermatology, immunology, endocrinology, ophthalmology and others. Hence, the presence of eyelid edema in patients who have not undergone surgical or minimal-invasive interventions to the periorbital area should prompt the physician to perform additional diagnostic measures (for a review, refer to [26]). Here, we present a retrospective analysis of 20 patients with eyelid edema successfully treated with the injection of hyaluronidase (Hylase Dessau®).

## Methods

We performed a retrospective analysis of 20 patients (Caucasian, 3 male, 17 female, ages 32 to 74 years, mean age 49.3 years) with lower eyelid edema treated with hyaluronidase (Hylase Dessau®). Fourteen patients presented with edema following tear-trough augmentation and six patients presented with eyelid edema of other origin (so called malar edema or malar mounds) (Table 1).

**Table 1:** Characteristics of the patients

**Cause of edema**	**Patients (*n*)**	**Male (*n*)/female (*n*)**	**Mean age (years)**	**Recurrence (%)**
Hyaluronan injection	14	0/14	50.2	0
Idiopathic	6	3/3	42.6	100

Prior to injection 150 IU of hyaluronidase (Hylase Dessau®) were dissolved in 1.0 ml of saline solution (0.9% NaCl). We performed local infiltration (micro-droplet technique) of approximately 0.2 to 0.5 ml of dissolved hyaluronidase (Hylase Dessau® 20 IU to 75 IU) per side directly into the edema using a 32 G injection needle after obtaining informed consent. Notably, the injected volume of hyaluronidase should not exceed the estimated volume of edema to avoid overcorrection. Photographs were taken before and one week after injection of hyaluronidase. For three patients, two sessions were required to achieve satisfactory results.

## Results

**Figure 1:**
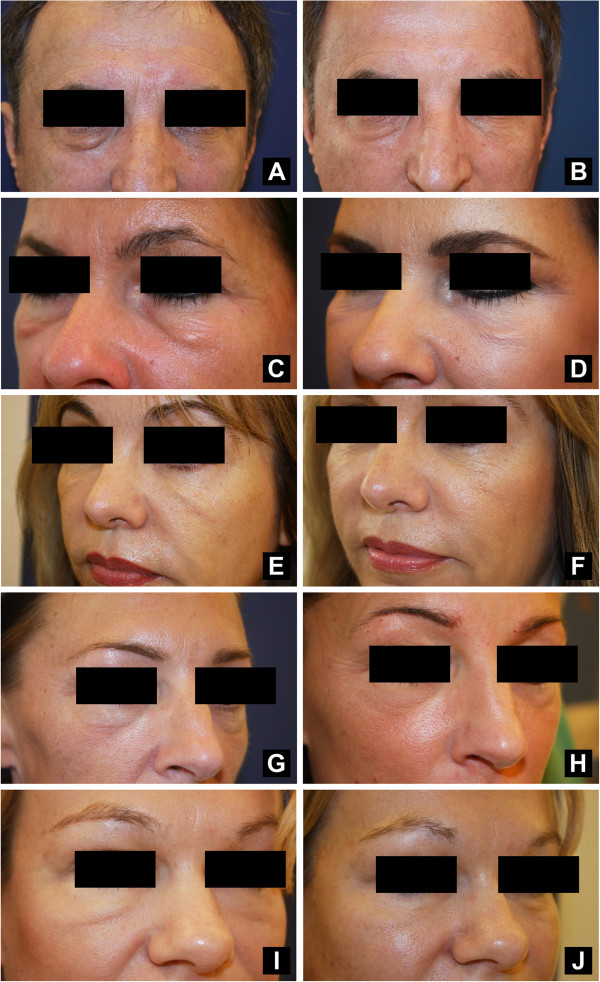
**Hyaluronidase injection is effective in the management of eyelid edema.** A 62-year-old man with primary lid edema **(A)** before and **(B)** one week after the injection of 0.5 ml (75 IU) of hyaluronidase into the right lower eyelid. A 46-year-old woman with primary lid edema **(C)** before and **(D)** after two weekly injections of 0.5 ml (75 IU) of hyaluronidase into each lower eyelid (total: 1.0 ml or 150 IU of hyaluronidase per side). A 50-year-old woman with lid edema following HA-filler injection **(E)** before and **(F)** one week after the injection of 0.5 ml (75 IU) of hyaluronidase into each lower eyelid. A 41-year-old woman with lid edema following HA-filler injection **(G)** before and **(H)** one week after the injection of 0.5 ml (75 IU) of hyaluronidase into each lower eyelid. A 46-year-old woman with lid edema following HA-filler injection **(I)** before and **(J)** one week after the injection of 0.5 ml (75 IU) of hyaluronidase into each lower eyelid.

The injection of approximately 0.2 to 0.5 ml of hyaluronidase (Hylase Dessau® 20 IU to 75 IU) was found to reduce effectively and rapidly or resolve eyelid edema after a single injection in the vast majority of treated patients (Figure 1A–F). For three patients, one additional injection was required to achieve satisfactory results. No adverse effects were observed except for expected needle-related bruising in some cases. However, in two cases we noticed that the hyaluronidase injection can be very powerful and it partly or completely dissolved the injected HA fillers, most likely due to the injection of a too large volume. We prefer to inject hyaluronidase gradually in small volumes at weekly intervals to prevent overtreatment, which may result in concave deformities. The images displayed are representative of the 20 cases.

While the effects of treatment of edema due to tear-trough augmentation were permanent, malar edema and malar mounds recurred within two to three weeks. Patients who had developed edema due to tear-trough augmentation did not receive any further HA injections in this area.

## Discussion

Tear-trough augmentation with HA fillers is a very effective minimal invasive esthetic procedure of increasing popularity. However, it must be noted that the periorbital and infra-orbital region is regarded as a high-risk area for injections. Blindness, which must be considered the most serious adverse event of periorbital filler injections, has been reported in only a few cases [16,27]. Edema are much more frequent and may occur in up to 26% of patients following tear-trough augmentation with HA fillers [23].

Hyaluronidase is regarded as the treatment of choice for the management of complications associated with HA fillers [14]. In an animal study, Kim *et al*. demonstrated that the early injection of hyaluronidase (four hours after filler injection) could significantly reduce the size of necrotic areas of rabbit ears, following injection of HA fillers into the auricular arteries compared to delayed injection of hyaluronidase (24 hours after filler injection) or untreated controls. The authors concluded that physicians should be aware of the potential risks of HA fillers. An intra-arterial injection may cause pain, a change in skin color or necrosis. The early injection of hyaluronidase can reduce these complications [28]. A retrospective analysis of complications following tear-trough augmentation with HA fillers for 100 patients by Morley *et al*. reported the use of post-interventional hyaluronidase injections in 7% of the patients [23].

In our experience, lid edema occurred in 10 to 70% of treated patients, depending on the injected HA filler. In this context, the frequency and level of edema are directly correlated to the water-binding capacity of the product. In most cases, HA fillers with a high water-binding capacity, e.g. certain volume fillers, should be avoided. Nevertheless, the use of modern volume fillers with lower water-binding capacities may be necessary for more severe defects. HA fillers associated with a high frequency of ‘blue lines’ should not be used for tear-trough augmentation, as the bluish aspect may be very disfiguring, especially for patients with thin skin. We suggest the following recommendations for the management of eyelid edema following tear-trough augmentation with HA fillers:

•The injected volume of hyaluronidase should match the estimated volume of the edema.

•Consider multiple treatment sessions with smaller volumes to avoid affecting the injected HA filler.

•An overdose may not only affect the injected HA filler but also the body’s own HA.

•Early interventions (up to a few weeks after development of edema) result in favorable responses (only one treatment session may be needed in most cases).

•Long-lasting edema (>six months) will likely require multiple (up to three) treatment sessions.

## Conclusions

In summary, eyelid edema can be regarded as a frequent complication of tear-trough augmentation. Infiltration of hyaluronidase is a rapid, safe and, in our opinion, currently the only effective option for the management of eyelid edema following HA-filler injections and may represent an interesting option for the treatment of eyelid edema of other origin. Additional studies are needed to validate our results for larger patient groups.

## Abbreviation

HA: hyaluronic acid or hyaluronan.

## Competing interests

This analysis was supported by Riemser Pharma GmbH. SH has received honoraria for advisory board meetings from Riemser Pharma GmbH. PAG has received research funding and honoraria for presentations and advisory board meetings from Riemser Pharma GmbH.

## Authors’ contributions

PAG and SH conceived the study, and participated in its design and coordination and helped to draft the manuscript. EB, BB and HS helped to draft the manuscript. All authors read and approved the final manuscript.
